# Anesthetic Management and Bispectral Index in a Child with Miller–Dieker Syndrome: A Case Report

**DOI:** 10.3390/children10040631

**Published:** 2023-03-28

**Authors:** Sang Jin Park, Jongyoon Baek, Suyoun Chun, Eun Kyung Choi

**Affiliations:** Department of Anesthesiology and Pain Medicine, Yeungnam University College of Medicine, Daegu 42415, Republic of Korea

**Keywords:** Miller–Dieker syndrome, lissencephaly, bispectral index, difficult airway, epilepsy

## Abstract

Miller–Dieker syndrome (MDS) is a genetic disorder characterized by classic lissencephaly, distinctive facial features, intellectual disability, seizures, and early death. The anesthetic management of patients with MDS should focus on airway manipulation with the risk of potentially difficult intubation, seizure control due to lissencephaly, and any other clinical complications. Herein, we report a case of anesthetic management in a child with MDS and describe relevant clinical features in a perioperative anesthetic setting. This case highlights the importance of difficult airway manipulation using a videolaryngoscope, seizure management with regard to anesthetics use, and the low validity of BIS monitoring in patients with MDS.

## 1. Introduction

Miller–Dieker syndrome (MDS) is a rare genetic disorder characterized by classic lissencephaly, distinctive facial features, intellectual disability, seizures, and early death [1]. The anesthetic management of patients with MDS should focus on airway manipulation with the risk of potentially difficult intubation, seizure control due to lissencephaly, and any other clinical complications. Previously, Wakiguchi et al. described the comprehensive anesthetic management in an 18-month-old girl with MDS [2]. Herein, however, we report a case of general anesthesia in a child with MDS, which specifically focuses on careful airway management using a videolaryngoscope, seizure management with regard to anesthetics use, and the validity of BIS monitoring. Relevant clinical features are described in a perioperative anesthetic setting.

## 2. Case Presentation

**Figure 1:**
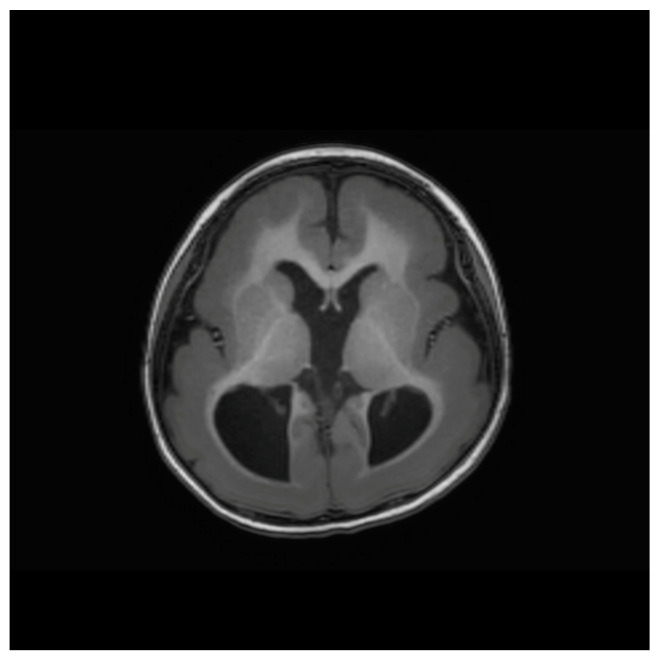
Brain magnetic resonance image (MRI) showing lissencephaly.

A 4-year-old girl (height, 95 cm; weight, 14 kg) previously diagnosed with Miller–Dieker syndrome was scheduled for intermittent exotropia surgery. A female infant (weighing 2510 g (23rd percentile), with a head circumference of 31 cm (9th percentile) and height of 47.5 cm (49th percentile)) was born at 37 weeks of gestation via cesarean section. Head ultrasonography showed ventriculomegaly, and brain magnetic resonance imaging revealed lissencephaly (Figure 1). A gene study confirmed a lissencephaly type 1 mutation, indicating a deletion in chromosome 17p13.3, including the PAFAH1B1 gene. Neonatal echocardiography showed a patent foramen ovale, which is a type of atrial septal defect. No other congenital anomalies were noted. Preoperative echocardiography showed patent foramen ovale occlusion. She developed a generalized tonic–clonic seizure from four months of age and was started on anticonvulsants (valproic acid and levetiracetam). She has been seizure-free since experiencing her last seizure a year before surgery.

The airway was evaluated prior to anesthesia. Her soft palate was intact and the Mallampati score was class 1. However, she showed a typical facial appearance of MDS: a short neck, prominent forehead, sunken appearance in the middle of the face, short nose, micrognathia, and thick upper lip. In the pre-anesthetic examination, she had a normal sinus rhythm on electrocardiography (ECG) with no murmur, and an echocardiogram showed no evidence of valve dysfunction or shunt disease with normal ejection pressure. Chest radiography revealed no distinctive abnormalities; chest auscultation was clear. The patient was prescribed anticonvulsants, oral valproic acid, and levetiracetam.

**Figure 2:**
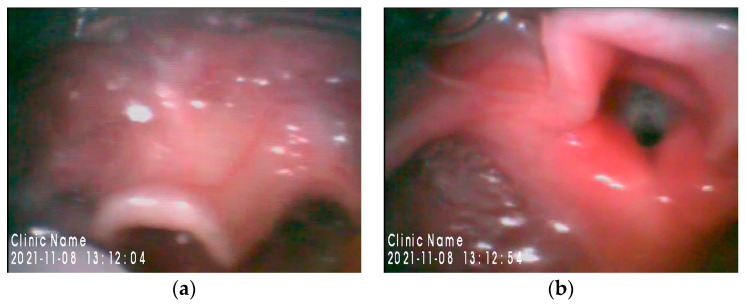
(**a**) Photograph of epiglottis on a Glidescope^®^ videolaryngoscope. (**b**) Laryngoscopic view of the glottis.

An experienced anesthesiologist performed video laryngoscopy for the problems associated with difficult intubation. Pulse oximetry (SpO_2_), ECG, and blood pressure (BP) were monitored in the operating room. We attached bispectral index (BIS, Aspect Medical Systems, Natick, MA, USA) monitoring on her forehead and train-of-four (TOF) monitoring on her left hand. Before the anesthesia, her heart rate was 127/min, BP was 104/71 mmHg, and SpO_2_ was 99%. In an awake state, BIS value was 37. Anesthesia was induced with thiopental sodium 75 mg and rocuronium 10 mg, and she was intubated with a 4.5 cuffed endotracheal tube using a Glidescope^®^ videolaryngoscope (Verathon Medical, Bothell, WA, USA). The epiglottis was large and curvilinear in the laryngoscopic view (Figure 2a), and intubation was uneventful. Laryngoscopic (Cormack-Lehane) grade was 2 (Figure 2b). Anesthesia was maintained with sevoflurane in 50% oxygen and remifentanil 0.1 mcg/kg/min. During anesthesia, the BIS values were between 17 and 26. Postoperatively, neuromuscular blockade was reversed with pyridostigmine and glycopyrrolate. Tracheal extubation was performed when her TOF was four and BIS score was 51. Her vital signs were stable in the recovery room.

## 3. Discussion

A child with MDS and a deletion at chromosome 17p13.3 had characteristic facial dysmorphism. Our patient had a short neck, prominent forehead, short nose, a protuberant upper lip, and micrognathia. These features can make airway management difficult. General predictors of difficult intubation include limited neck extension and mouth opening, macroglossia, micrognathia, and structural abnormalities in the laryngotracheal passage. Securing an airway in pediatric anesthesia practice is more challenging due to anatomical and physiological differences compared to adults with prominent occiput, higher larynx and U-shaped epiglottis, relatively greater oxygen consumption, and somewhat lower functional residual capacity [3]. In particular, the clinical assessment of syndromic children with craniofacial abnormalities should focus more on difficulty with face mask ventilation and endotracheal intubation, or both. Congenital cardiac lesions, seizure disorders, and developmental delay should also be carefully considered in difficult airway management. Thus, setting up special equipment for difficult airways should be considered. An anesthesiologists must pay close attention to the above-mentioned issues. Wakiguchi et al. reported a case of airway management in an 18-month-old girl with MDS underwent laryngotracheal separation surgery [2]. In that case, a direct laryngoscope with a Macintosh blade was used. However, we used a videolaryngoscope for tracheal intubation, even though the Mallampati grade was 1 in the preoperative evaluation. The reliability of Mallampati grade in predicting a difficult airway in children may be questionable; therefore, we prepared equipment for identifying this, including oropharyngeal airway, correctly sized facemask, pediatric video laryngoscopy, and fiberoptic bronchoscopy preoperatively. Tracheal intubation was uneventful with Glidescope^®^ videolaryngoscope; however, careful airway management was required because of an increased risk of aspiration and gastroesophageal reflux in patients with MDS. Here, the use of laryngeal mask airway (LMA) can be considered as an alternative to tracheal intubation. Difficult intubation is more frequent in children with congenital craniofacial anomalies; therefore, LMA insertion should be prepared for children with known or suspected difficult airways [4].

Another anesthetic concern in children with MDS is related to seizure management. Most of these children have a significant history of seizures. Seizures can be seen in those with developmental or structural brain abnormalities as a result of an imbalance between excitatory and inhibitory neuronal activities. Many anesthetic agents affect the propensity to seizure; thus, both the appropriate perioperative control of seizure incidence and awareness of potential epileptogenic anesthetic agents are important in patients with MDS. Some studies reported that sevoflurane provokes seizure-like activities, particularly in children [5], whereas intravenous anesthetic agents, such as propofol and barbiturates show anticonvulsant properties at anesthetic doses [6,7]. However, intravenous anesthetic agents are not recommended for the maintenance of anesthesia because BIS monitoring is hampered in patients with MDS. According to a report by Valkenburg et al., BIS values had a range of 14–22 in a 2-year-old awake child with West syndrome and lissencephaly [8]. They speculated that low BIS values might be a consequence of abnormal EEG characteristics. Additionally, Wachiguchi et al. reported a case of MDS in an 18-month-old child. The child had BIS values of 16–21 before anesthetic induction, 15–34 during sevoflurane maintenance, and 26–33 after anesthetic administration [2]. In our case, the BIS values were 37, 17–26, and 51 before, during, and after the anesthesia, respectively. Although BIS values in children aged >1 year are correlated with the depth of general anesthesia and are inversely proportional to end-tidal sevoflurane [9], in children with MDS, BIS monitoring may not represent adequate information for anesthetic depth because they have an extremely low baseline BIS value in the awake state. With the exception of the consequences of EEG abnormalities, the questionable validity of BIS in children with MDS may be due to the anticonvulsant effect on the depth of anesthesia. Anesthetics (volatile and intravenous) and anticonvulsants both act in the gamma aminobutyric acid (GABA) receptor in the central nervous system. Except cases of syndromic children, unusually low BIS values have been seen during cerebral ischemia [10] and hydrocephalus [11]. The possible explanation of these low BIS values was suggested to be caused by decreased cerebral perfusion. In the MDS syndrome, except the effects of anticonvulsants, increased intracranial pressure due to lissencephaly and ventriculomegaly may have caused consequent cerebral hypoperfusion, which is associated low BIS values and EEG abnormalities.

The long-term use of anticonvulsants can affect the rapid metabolization of neuromuscular blockers and opioids by upregulating hepatic P450 enzymes [12]. Accordingly, intraoperative neuromuscular monitoring should be used, and respiratory monitors should also be used postoperatively due to risks of pulmonary complications.

Other reported congenital comorbidities of MDS include congenital heart disease (patient ductus arteriosus and atrial and ventricular septal defects), genitourinary anomalies (renal anomaly and cryptorchism), gastrointestinal anomalies (omphalocele), and limb anomalies (contracture and polydactyly) [13]. Anesthesiologists should focus on all possible clinical disorders and warrant detailed management.

In conclusion, we present a case of general anesthesia in a child with MDS, which specifically focuses on difficult airway, seizure management, and the validity of BIS monitoring. Therefore, anesthesiologists should be concerned about these issues in the anesthesia of MDS patients.

## Data Availability

Not applicable.
